# Supplementary material for: Analysis of 16S rRNA Gene Sequence of Nasopharyngeal Exudate Reveals Changes in Key Microbial Communities Associated with Aging
Source: Int J Mol Sci. 2023 Feb 18;24(4):4127. doi: 10.3390/ijms24044127 (PMC9960676; doi:10.3390/ijms24044127)
Supplement: Supplementary file 1 [file ijms-24-04127-s001.zip › ijms-2182098-supplementary.pdf]

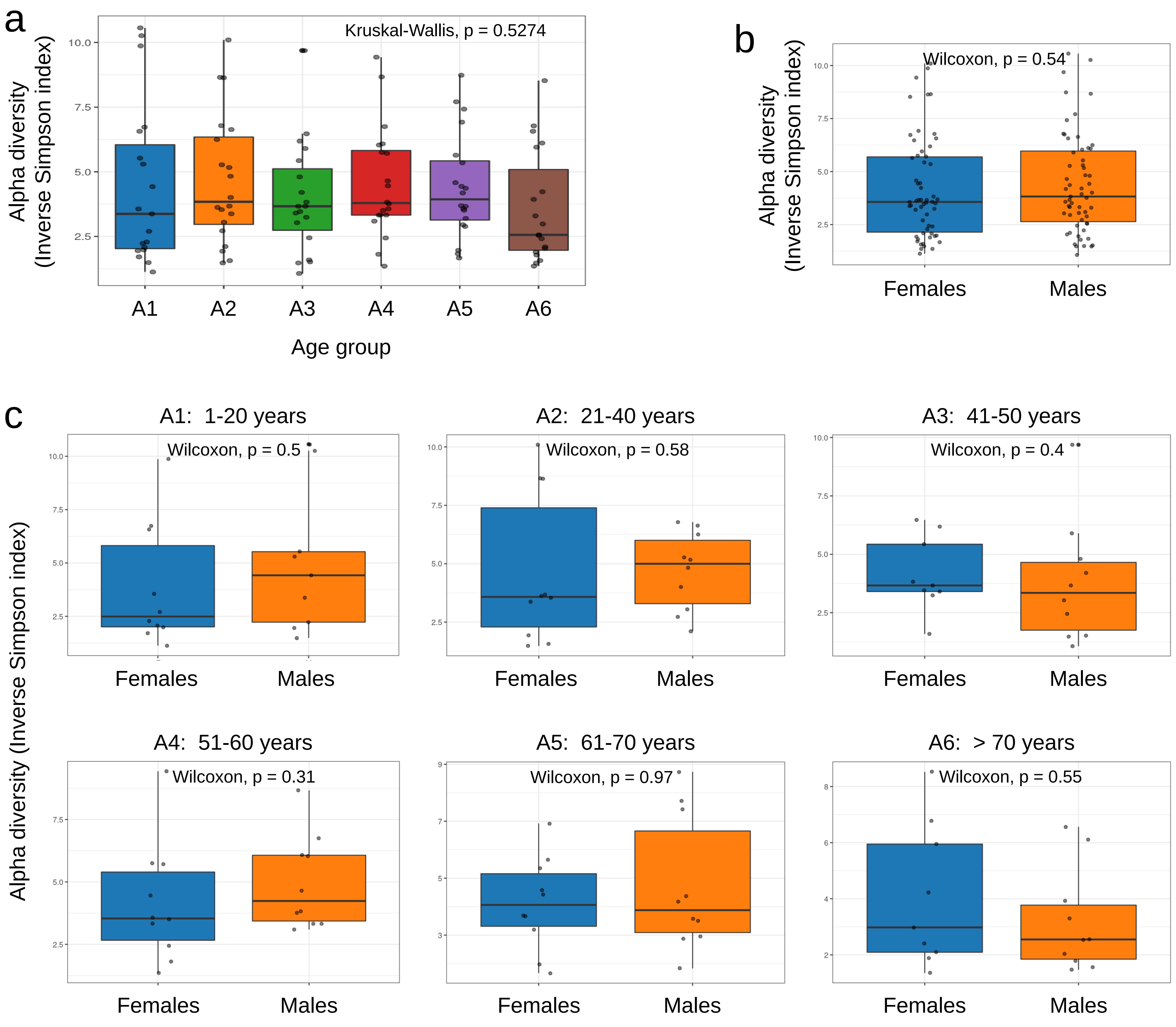

Figure S1. Comparison of alpha diversity parameters across the age and sex groups studied. Box-whisker plots of the alpha diversity inverse Simpson index and its comparison using the Kruskal-Wallis test among the different age groups established for this study (**a**), and the Wilcoxon signed-rank test between females and males (**b-c**). Each sample is represented by one dot.

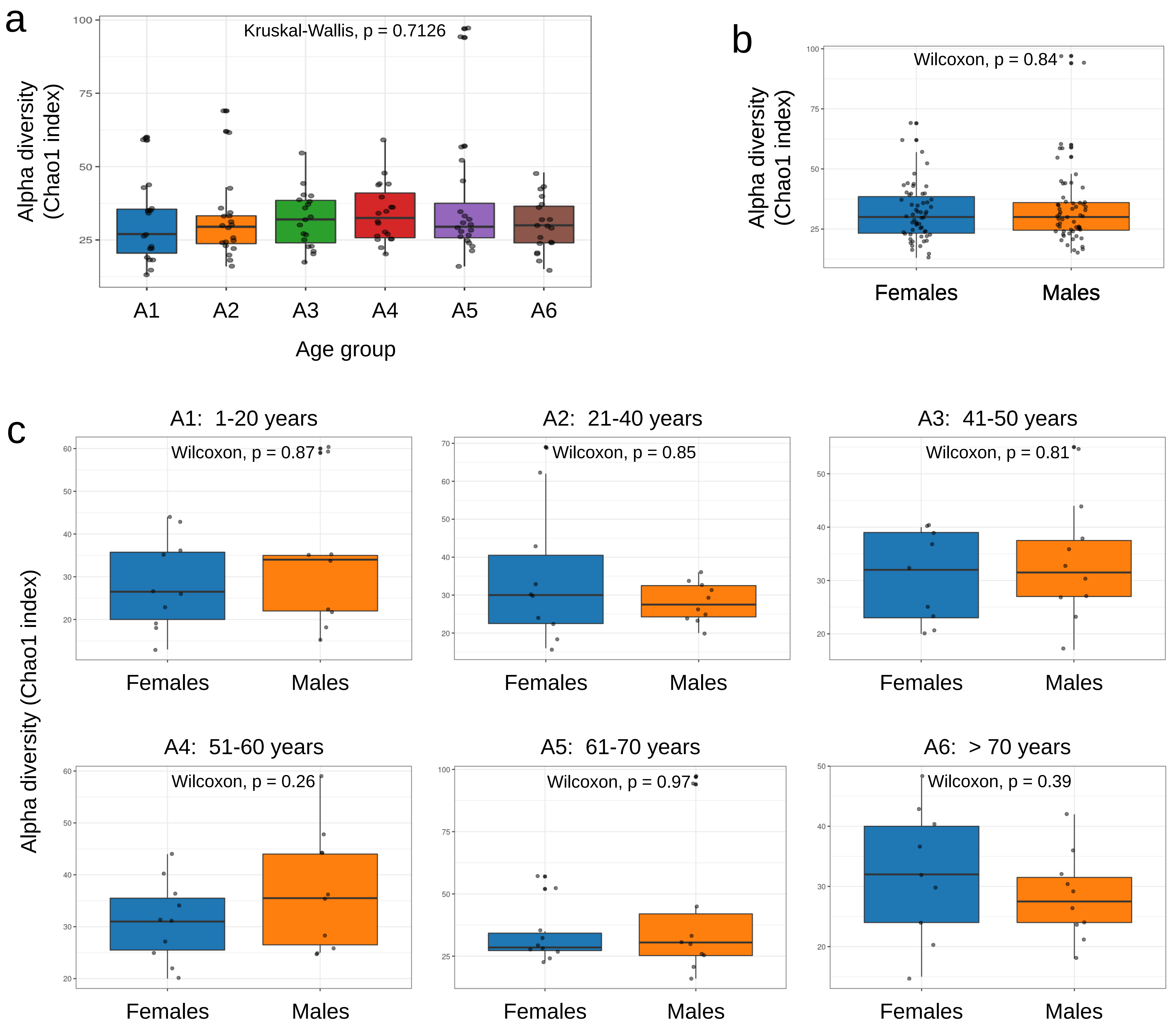

Figure S2. Comparison of alpha diversity parameters across the age and sex groups studied. Box-whisker plots of the alpha diversity Chao1 index and its comparison using the Kruskal-Wallis test among the different age groups established for this study (**a**), and the Wilcoxon signed-rank test between females and males (**b-c**). Each sample is represented by one dot.

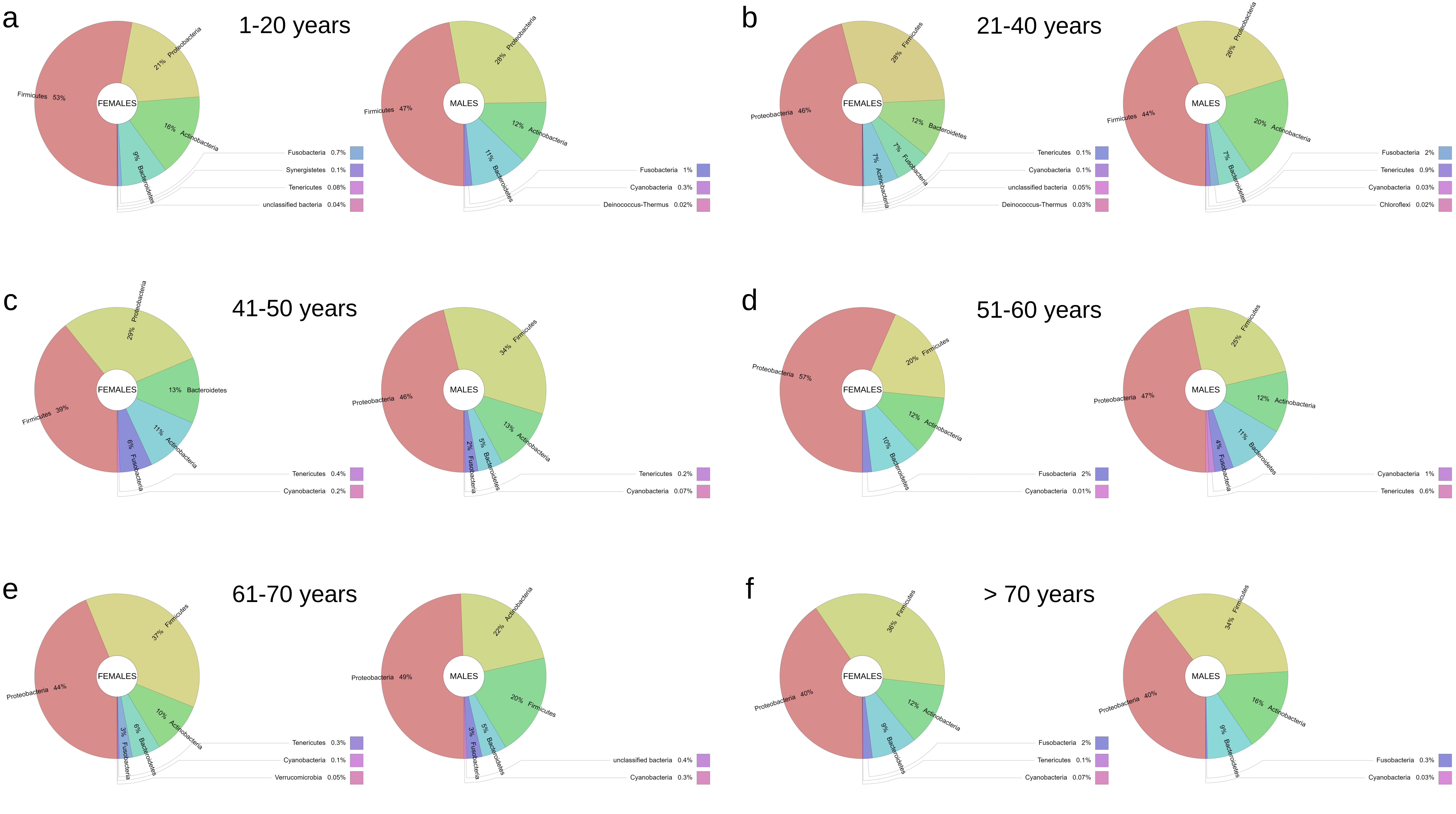

Figure S3. Taxonomic composition and age- and sex-associated metagenomic changes in the nasopharynx of healthy people. a-f Krona charts showing bacterial community composition at phylum level in the indicated age and sex groups.

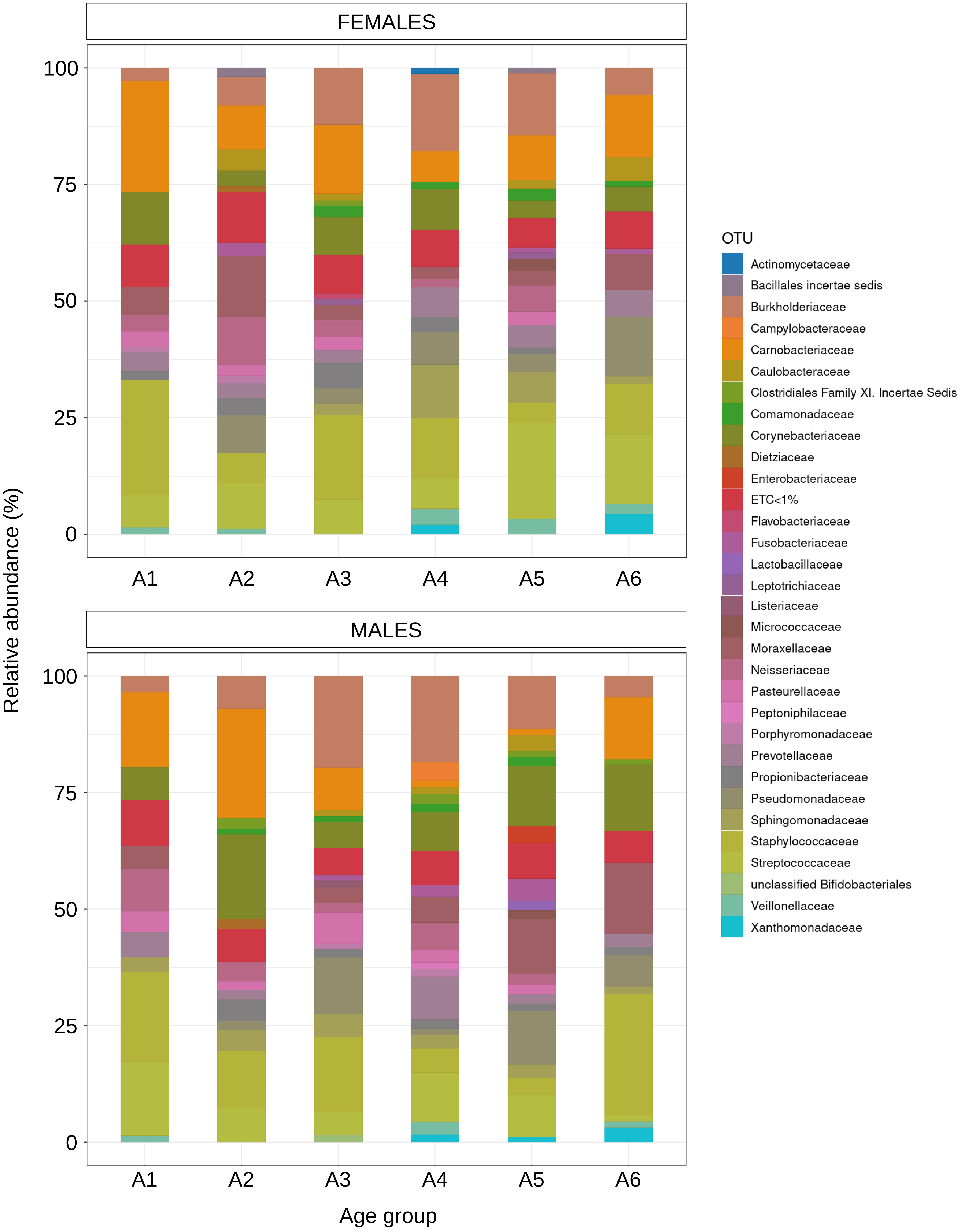

Figure S4. Taxonomic composition and age- and sex-associated metagenomic changes at the family level in the nasopharynx of healthy people. Stacked bar charts showing the relative abundance (%) of bacterial families in the indicated age groups and separately by sex. For clarity, only the bacterial families with average abundance > 1% at each age group are shown.

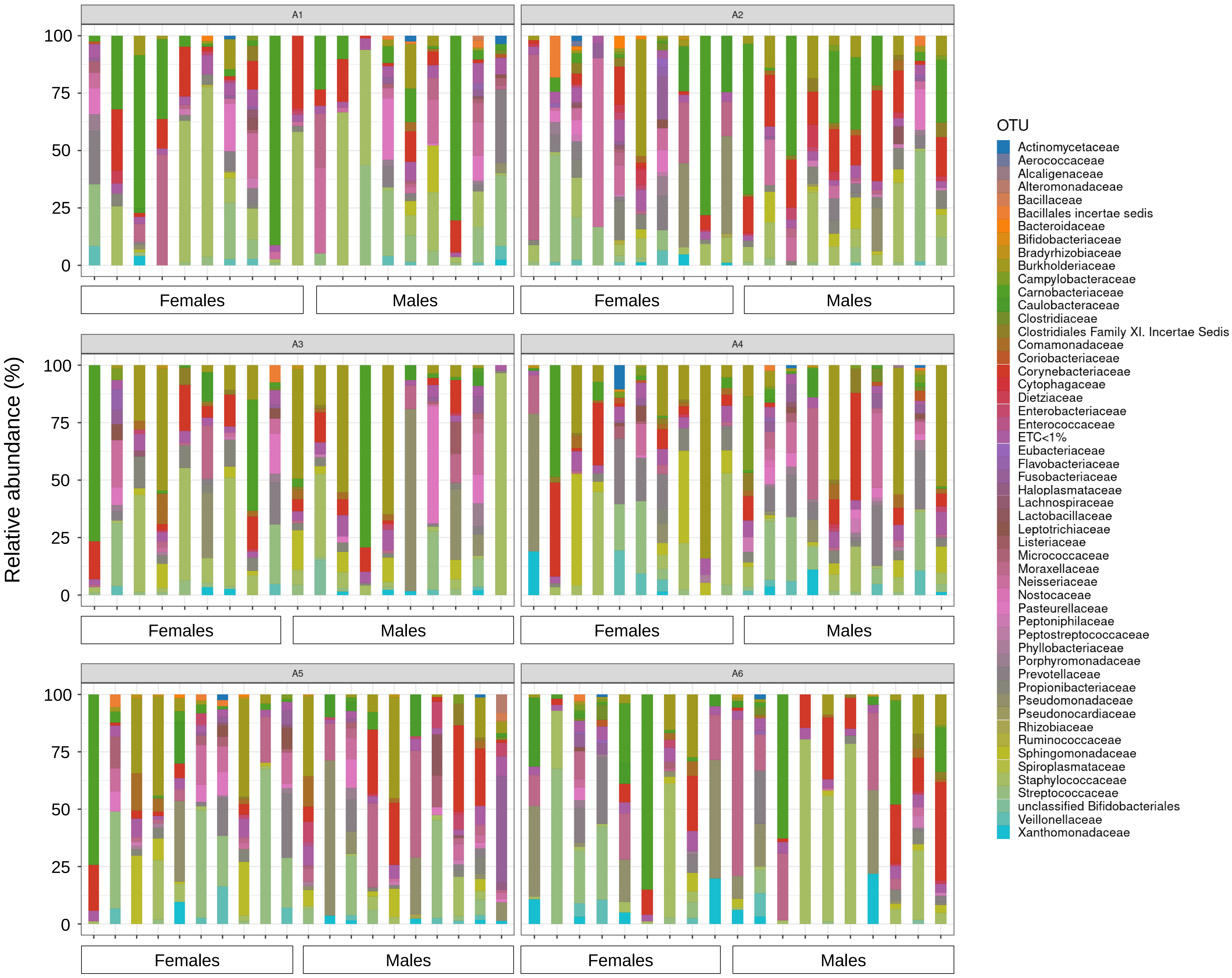

Figure S5. Taxonomic composition and age- and sex-associated metagenomic changes at the family level in the nasopharynx of each individual included in this study. Stacked bar charts showing the relative abundance (%) of bacterial families, in the indicated age groups and separately by sex, of each subject included in this study. For clarity, only the bacterial families with average abundance > 1% at each age group are shown.

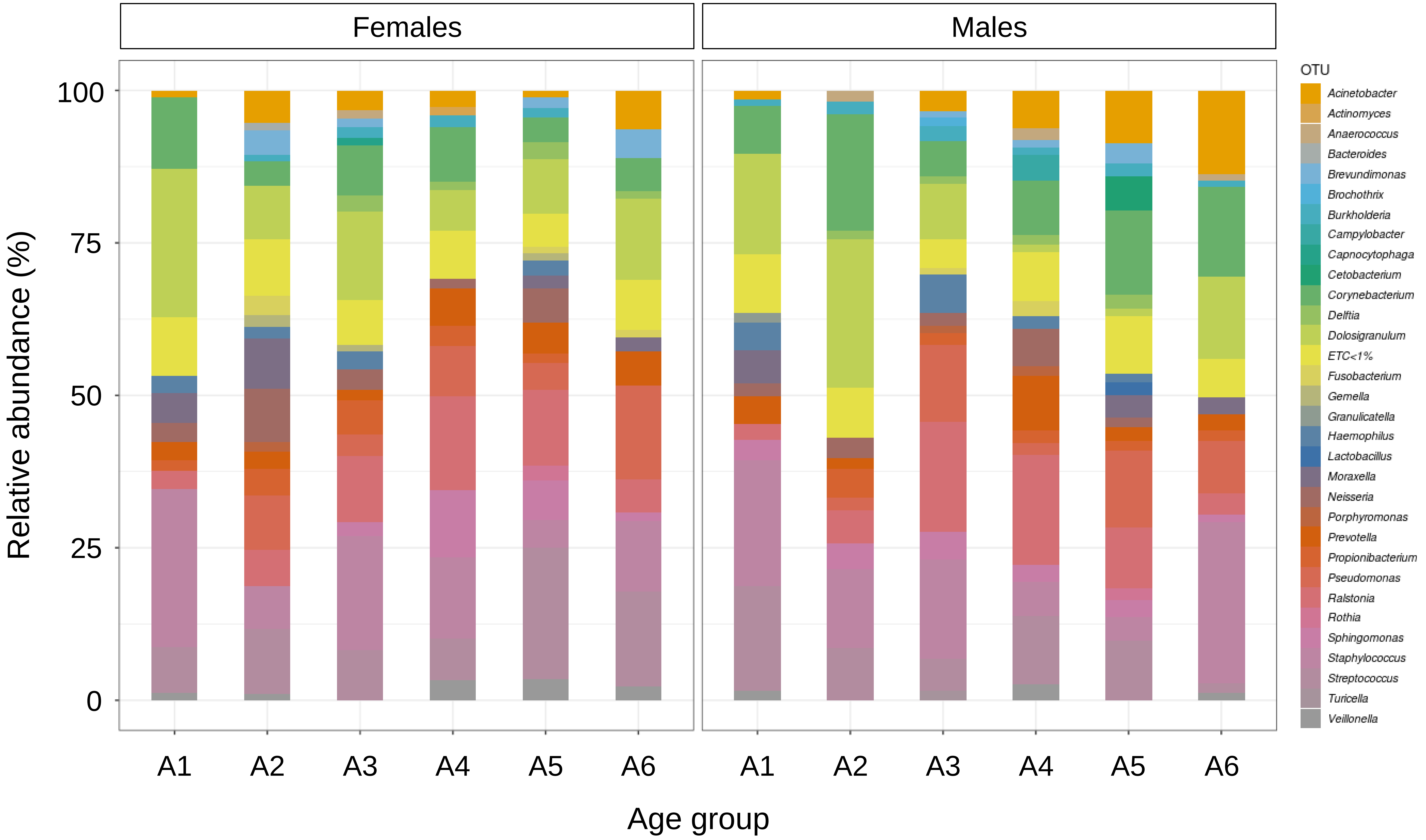

Figure S6. Taxonomic composition and age- and sex-associated metagenomic changes at the genus level in the nasopharynx of healthy people. Stacked bar charts showing the relative abundance (%) of bacterial genera in the indicated age groups and separately by sex. For clarity, only the bacterial genera with average abundance > 1% at each age group are shown.

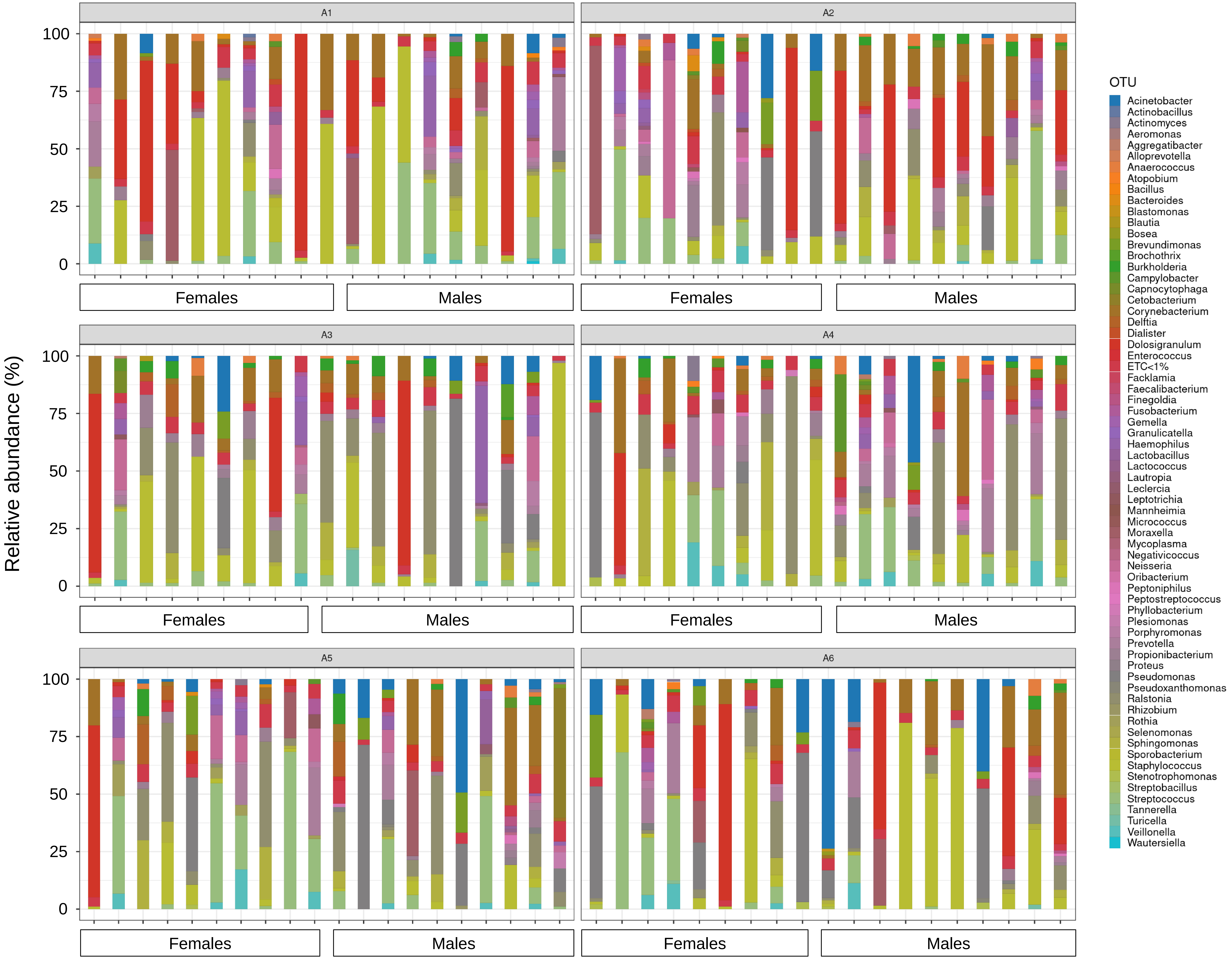

Figure S7. Taxonomic composition and age- and sex-associated metagenomic changes at the genus level in the nasopharynx of each individual included in this study. Stacked bar charts showing the relative abundance (%) of bacterial genera, in the indicated age groups and separately by sex, of each subject included in this study. For clarity, only the bacterial genera with average abundance > 1% at each age group are shown.

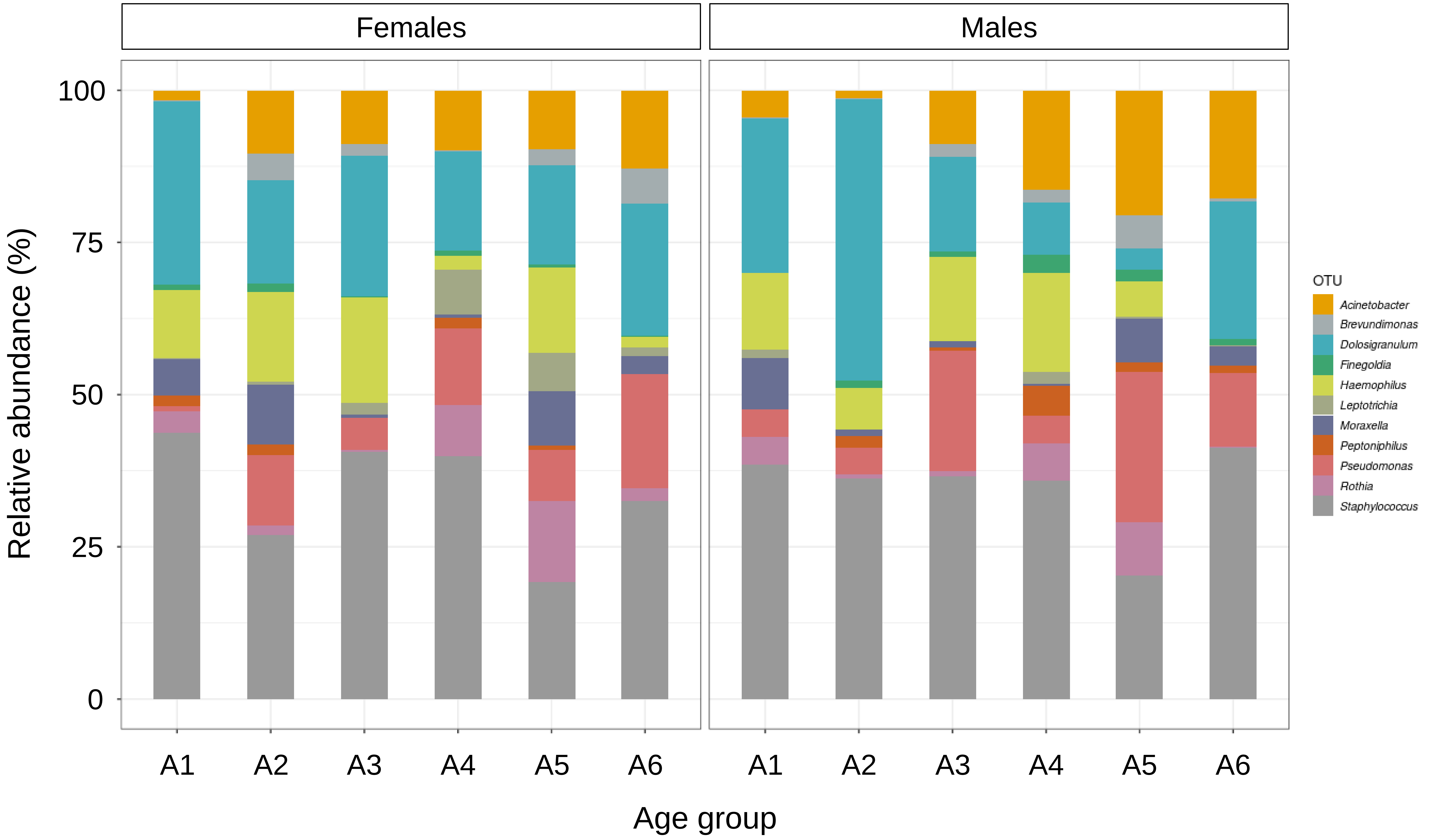

Figure S8. Taxonomic composition and age- and sex-associated metagenomic changes at the genus level in the nasopharynx of healthy people. Stacked bar charts showing the relative abundance (%) of the 11 selected bacterial genera indicated, in the indicated age groups and separately by sex.

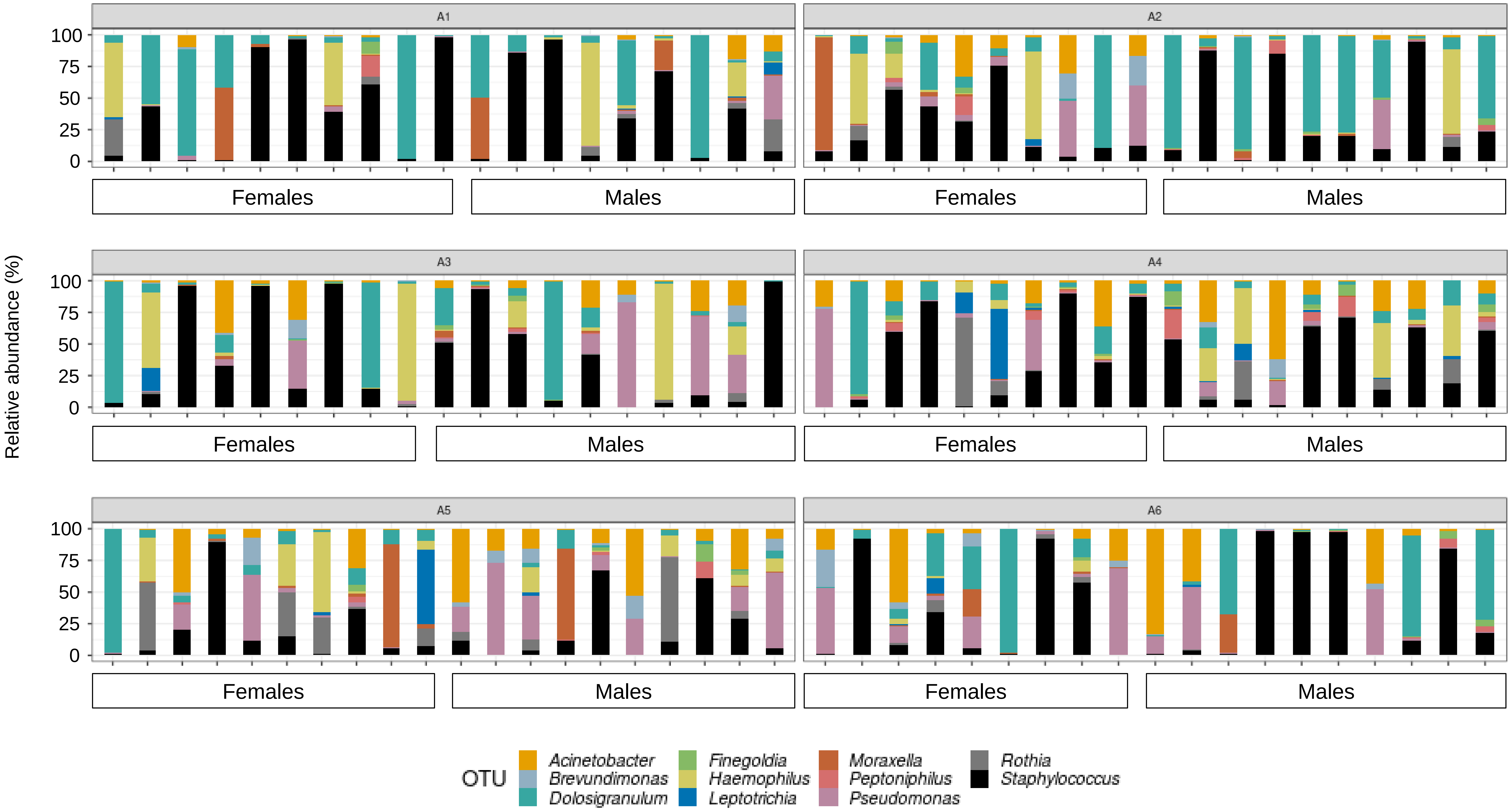

Figure S9. Taxonomic composition and age- and sex-associated metagenomic changes at the genus level in the nasopharynx of each individual included in this study. Stacked bar charts showing the relative abundance (%) of the 11 selected bacterial genera indicated, in the indicated age groups and separately by sex, of each subject included in this study. Note that the rest of bacterial genera are not taken into consideration in this analysis.

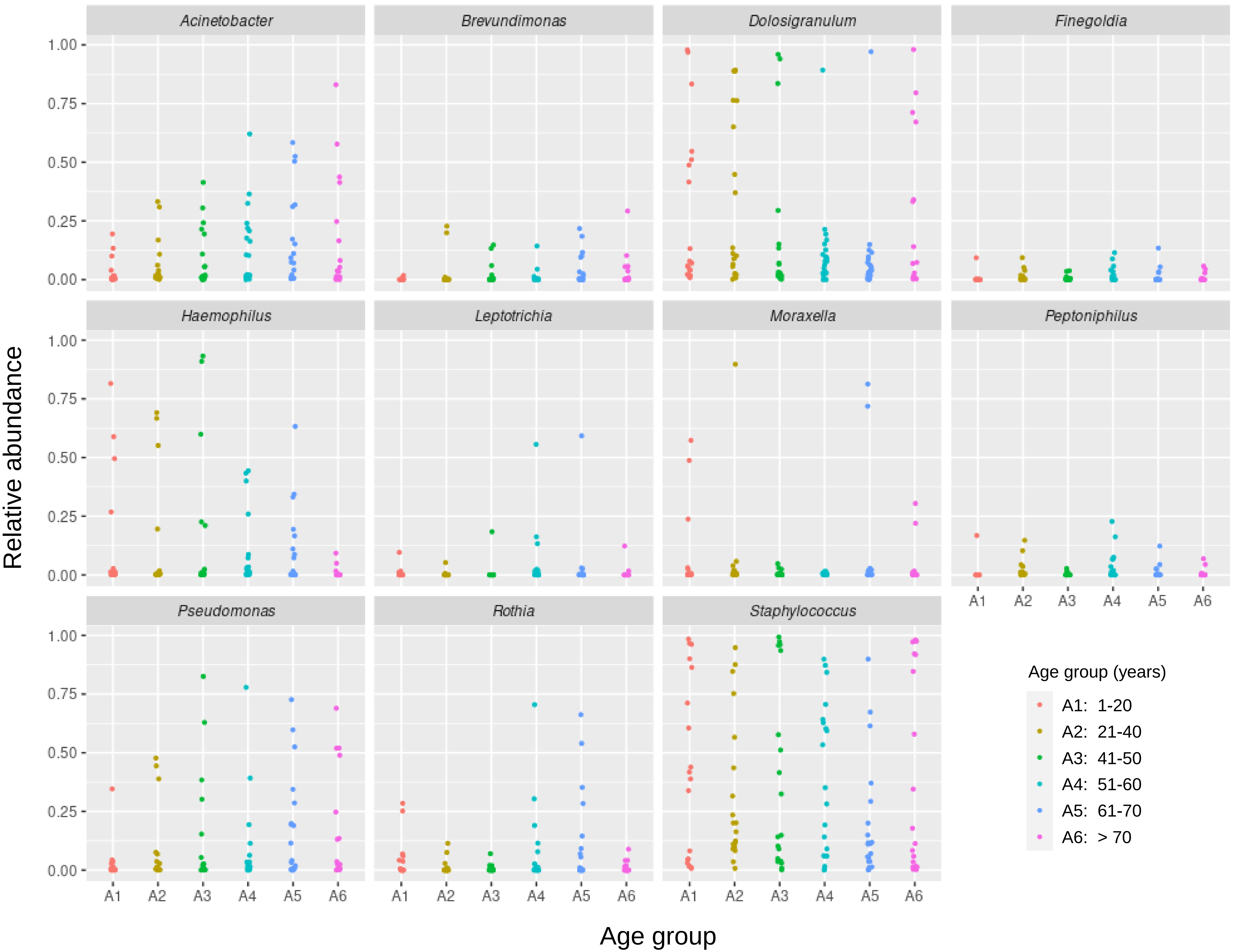

Figure S10. Relative abundance of the 11 bacterial genera which present significant differences between age groups in each individual. Relative abundance of the selected 11 bacterial genera indicated in each subject included in this study, in the indicated age groups. Each sample is represented by one dot, colored according to age.

Table S1. Nasopharyngeal exudate samples collected from healthy (not infected with SARS-CoV-2) males and females in each age group

Age group	Sex	Samples
A1: 1-20 years	*Female*	10
	Male	9
A2: 21-40 years	*Female*	10
	Male	10
A3: 41-50 years	*Female*	10
	Male	10
A4: 51-60 years	*Female*	10
	Male	10
A5: 61-70 years	*Female*	10
	Male	10
A6: >70 years	*Female*	10
	Male	10
Total	*Female*	60
	Male	59

Table S2. Summary of the statistical analysis of the relative abundance differences among the 6 age groups established in this study. Only the statistically significant differences (adjusted p-value < 0.05) for the 11 bacterial genera which present such differences are shown

Genus	baseMean	log2FoldChange	lfcSE	stat	Adjusted p-value	Age groups compared
Acinetobacter	848,877	-2,463	0,664	-3,71	0,009	A1_A6
		-2,866	0,655	-4,374	0,003	A2_A6
		2,378	0,655	3,629	0,02	A3_A4
		-3,688	0,655	-5,628	0	A4_A6
		-2,338	0,655	-3,57	0,019	A5_A6
Brevundimonas	129,952	-5,191	1,366	-3,799	0,01	A1_A2
		-5,772	1,383	-4,173	0,002	A1_A6
Dolosigranulum	16684,133	5,033	0,977	5,154	0	A1_A4
		3,581	0,964	3,714	0,021	A2_A4
		4,244	0,977	4,346	0,001	A3_A4
		-4,553	0,977	-4,662	0	A4_A6
Finegoldia	50,796	-5,379	1,527	-3,523	0,022	A1_A2
Haemophilus	450,095	3,981	0,961	4,141	0,002	A1_A6
		4,733	0,961	4,925	0	A3_A6
		4,461	0,949	4,701	0	A5_A6
Leptotrichia	21,087	8,756	2,111	4,148	0,005	A1_A3
		-10,723	2,08	-5,156	0	A3_A4
		-10,303	2,08	-4,953	0	A3_A5
		-7,858	2,112	-3,72	0,021	A3_A6
Moraxella	90,658	3,938	0,991	3,973	0,004	A1_A4
Peptoniphilus	55,534	-7,681	1,739	-4,416	0,001	A1_A2
		-7,135	1,739	-4,103	0,003	A1_A4
		-6,649	1,761	-3,776	0,008	A1_A6
Pseudomonas	2779,023	-4,48	0,933	-4,799	0	A1_A2

		-3,823	0,945	-4,044	0,005	A1_A3
		-5,261	0,945	-5,566	0	A1_A6
		3,676	0,921	3,994	0,014	A2_A4
		-4,458	0,933	-4,781	0	A4_A6
Rothia	115,404	-5,551	1,281	-4,332	0,003	A2_A5
		-4,724	1,294	-3,65	0,027	A3_A5
		5,027	1,296	3,878	0,007	A5_A6
Staphylococcus	8272,167	2,477	0,721	3,434	0,025	A1_A2
		2,635	0,73	3,608	0,021	A1_A3
		3,539	0,721	4,908	0	A1_A4
		4,078	0,721	5,656	0	A1_A5
		-2,865	0,721	-3,974	0,004	A4_A6
		-3,405	0,721	-4,722	0	A5_A6
